# Legal Information Retrieval and Entailment Using Transformer-based Approaches

**DOI:** 10.1007/s12626-023-00153-z

**Published:** 2024-01-11

**Authors:** Mi-Young Kim, Juliano Rabelo, Housam Khalifa Bashier Babiker, Md Abed Rahman, Randy Goebel

**Affiliations:** 1 Department of Science, Augustana Faculty, University of Alberta, Camrose, Alberta Canada; 2 Alberta Machine Intelligence Institute, University of Alberta, Edmonton, Alberta Canada; 3 Department of Computing Science, University of Alberta, Edmonton, Alberta Canada; 4 Department of Computing Science and Alberta Machine Intelligence Institute, University of Alberta, Edmonton, Alberta Canada

**Keywords:** COLIEE 2023, Legal information retrieval, Legal information entailment, Transformer-based legal information extraction

## Abstract

The challenge of information overload in the legal domain increases every day. The COLIEE competition has created four challenge tasks that are intended to encourage the development of systems and methods to alleviate some of that pressure: a case law retrieval (Task 1) and entailment (Task 2), and a statute law retrieval (Task 3) and entailment (Task 4). Here we describe our methods for Task 1 and Task 4. In Task 1, we used a sentence-transformer model to create a numeric representation for each case paragraph. We then created a histogram of the similarities between a query case and a candidate case. The histogram is used to build a binary classifier that decides whether a candidate case should be noticed or not. In Task 4, our approach relies on fine-tuning a pre-trained DeBERTa large language model (LLM) trained on SNLI and MultiNLI datasets. Our method for Task 4 was ranked third among eight participating teams in the COLIEE 2023 competition. For Task 4, We also compared the performance of the DeBERTa model with those of a knowledge distillation model and ensemble methods including Random Forest and Voting.

## Introduction

Every day, large volumes of legal data are produced by law firms, law courts, independent attorneys, legislators, regulators, and many others. Within that context, the disciplined management of legal information becomes manually intractable and requires the development of tools that automatically or semi-automatically aid legal professionals in managing information overload. The COLIEE competition^1^ addresses four facets of that challenge: case law retrieval, case law entailment, statute law retrieval, and statute law entailment. Here we summarize the details of our approach to case law retrieval and statute law entailment, evaluate the results achieved, and comment on future work to further improve our models.

The case law retrieval task (Task 1) involves identifying legal cases that should be “noticed” concerning a given query case from amongst a given set of candidate cases. In Task 1, our goal is to identify legal cases that are relevant to a given query case and that support the decision of the given query case. These relevant legal cases are referred to as “noticed cases.” in Task 1. Our approach to this challenging task relies on a transformer-based model that creates a multidimensional numeric representation of every paragraph within each case. We then calculate cosine distances between each paragraph of a query case and a candidate case, create a histogram from the results, and use those distances to train a binary classifier to determine whether an input case should be noticed. Additionally, in the context of the COLIEE datasets, we perform some simple pre-processing and post-processing steps, such as removing French fragments and applying a minimum confidence score for the classifier outputs, before generating the final results.

In the COLIEE competition, Task 4 focuses on legal entailment, which is to predict the entailment relationship between a legal query and statutory law articles, by comparing the meaning of the legal query and the law articles. In general, this task requires participants to develop algorithms capable of reading a query and a law article (or multiple law articles) and then determining whether the law article(s) entail the legal query or not. In other words, the goal of the statute law entailment (Task 4) is to construct yes/no question-answering systems for legal queries, by confirming entailment of a query from articles. The answer to a question is typically determined by measuring some kind of semantic similarity between question and answer. Because the legal bar exam query and articles are complex and varied, we need to carefully determine what kind of information is needed to confirm textual entailment. Here we exploit the idea of natural language inference and fine-tune a DeBERTa-large language model (LLM) to construct a yes/no question-answering system for legal queries.

Our approach for Task 4 relies on a transformer (DeBERTa)-based model to construct a classifier for yes/no questions. The DeBERTa model was initially trained for the natural language inference task (NLI) using two datasets, namely SNLI [2] and MultiNLI [35]. In addition, to standardize all the inputs to the model, we provide all lowercase sentences to the model to generate the final results. This approach achieved an accuracy of 0.6634 in the official test dataset, which was ranked third amongst eight competitors in Task 4 of the COLIEE 2023 competition.

Our paper is organized as follows: Sect. 2 presents a brief state-of-the-art review; Sects. 3.1 and 4 describe our method in more detail. Section 5 analyzes the results along with some subsequent experiments with smaller transformers and with models pre-trained on legal data. Section 5 also includes a fault analysis of some models. Finally, Sect. 6 provides some final remarks and proposes some future work.

## Literature Review

Most current approaches to legal information retrieval rely on traditional information retrieval (IR) methods and more recently, transformer-based large language model (LLM) techniques. Here we briefly summarize some of the most successful approaches in Task 1, proposed in recent editions of the COLIEE competition.

*TR* [30] uses a two-phase approach to legal case document information retrieval. In the first phase, they generate a candidate set optimized for recall, aiming to include all true noticed cases while removing some false candidates. In the second phase, they train a binary classifier to predict whether a given $(query\ case, candidate\ case)$ pair represents a true noticed relationship. In this step, these authors experimented with logistic regression, naive Bayes, and tree-based classifiers.

*NeuralMind* [29] applied “vanilla” BM25 to the case law retrieval problem. The authors first indexed all base and candidate cases in the dataset. Before indexing, each document was split into segments of text using a context window of 10 sentences with overlapping strides of 5 sentences (the “candidate case segments”). BM25 was then used to retrieve candidate case segments for each base case segment. The relevance score for a $(base\ case, candidate\ case)$ pair was the maximum score among all the base case and candidate case segment pairs. The candidates were then ranked using empirically determined threshold heuristics.

*TUWBR* [7] starts from two assumptions: first, that there is a topical overlap between a query and noticed cases, but that *not all parts of a query case are equally important*. Secondly, they assume that traditional IR methods, such as BM25, provide competitive results in Task 1. They perform both document-level and text-passage-level retrieval and also augment the system by adding external domain knowledge by extracting statute fragments and explicitly adding those fragments to the documents.

*JNLP* [3] applies an approach that first splits the documents into paragraphs, and then calculates the similarities between cases by combining term-level matching and semantic relationships at the paragraph level. An attention model is applied to encode the whole query in the context of candidate paragraphs, which provides the basis to infer the relationship between cases.

*DoSSIER* [1] combined traditional and neural network-based techniques in Task 1. The authors investigate lexical and dense first-stage retrieval methods aiming for a high recall in the initial retrieval and then compare shallow neural network re-ranking between the MTFT-BERT model and the BERT-PLI model. They then investigate which part of the text of a legal case should be taken into account for re-ranking. Their results show that BM25 shows a consistently high effectiveness across different test collections in comparison to the neural network re-ranking models.

Task 1 has been recently adjusted in COLIEE. The new configuration increased the difficulty so that previously used Information Retrieval methods, even augmented with transformer-based approaches, did not show great results [24]. Given that most of the current approaches work at the document level, we chose to experiment with the documents at the sentence level to try and capture more localized information. More details of the approach are presented in Sect. 3.1.

In the same manner, we briefly summarize some of the most successful approaches in Task 4, proposed in recent editions of the COLIEE competition.

*KIS* [9] adopted an ensemble approach that combines their rule-based method leveraging predicate-argument structures, with the incorporation of BERT-based methods. Their BERT-based methods include data augmentation, data selection, and person name inference. They showed the highest performance in the COLIEE 2022 competition.

*HUKB* [36] introduced a method for selecting relevant portions from articles, accompanied by a new data augmentation method. This was added by an ensemble approach that combined BERT with data augmentation and extraction of judicial decision sentences.

*JNLP* [3] conducted a comparative analysis among ELECTRA, RoBERTa, and Legal-BERT.

*LLNTU* [19] restructured the provided data into a dataset comprising disjunctive union strings derived from training queries and articles. They also developed a similarity comparison model based on the longest uncommon subsequence.

*OvGU* [34] utilized an ensemble of graph neural networks (GNNs), combined with referring textbook nodes and averaged sentence embeddings. The details of the COLIEE 2022 competition methods are in [17].

Deep learning methods have enabled the construction of complex and accurate models for the Natural Language Inference (NLI) of Task 4 ( [25, 28]). Most current approaches to the NLI problem are formulated as a 3-way classification (entailment, contradiction, and neutral) of the entailment relation between a pair of sentences. Properly approached, this task requires a sophisticated semantic framework to understand the context for two sentences (premise, hypothesis) ( [10, 26]). In other words, textual entailment is a logical reasoning task in which the goal is to determine whether one sentence can be inferred from another (more generally, whether one text segment can be inferred from another).

Deep nets have achieved encouraging performances on many NLI datasets such as [6, 18], and [23]. Most of related work employs complex neural architectures such as RNNs: [33, 20, 31], CNNs: [11], and BERT: [13]. In general, the improvements in performance suggest that these models tend to capture better representations from the datasets that can be used as proxies to predict the relationship between sentences. In the sentential case, the NLI task usually consists of classifying an ordered pair of sentences into one of three categories: “positive entailment” occurs when one can use the first sentence to prove that a second sentence is true. Conversely, “negative entailment” occurs when the first sentence can be used to disprove the second sentence. Finally, if the two sentences do not correlate, as determined by the failure of the first two tests, they are considered to have a “neutral entailment.”

The statute law entailment task (Task 4) in COLIEE is similarly designed: the participants are required to decide if a query is entailed from the civil law statutes.

NLI datasets are typically built by asking annotators to compose sentences based on premises extracted from existing corpora so that the composed sentences stand in entailment/ contradiction/ neutral relationship to the premise [16]. In COLIEE 2023, we have two relationships that need to be verified: entailment (yes) and non-entailment (no).

For this problem, we rely on the base DeBERTa model [13] which is an extension of the original BERT model. The DeBERTa-based model was trained on large volumes of raw text corpora using the idea of self-supervised learning. As compared to the original BERT model, DeBERTa captures more fine-grained contextual information and relationships between tokens, resulting in a significant performance gain on a wide range of Natural Language Understanding (NLU) tasks.

On the other hand, we also experimented with knowledge distillation approaches. Knowledge distillation (KD), refers to the process of compressing larger transformers into smaller versions that have similar performance to their larger counterparts. In this paper, we experimented with nli-MiniLM2-L6-H768 (MiniLMV2) [32] KD model, trained on the QNLI [35] and SNLI [2] datasets. In the MiniLMV2 paper [32], the authors used a teacher-student paradigm to train the smaller KD model, whereas a larger BERT-based model worked as the teacher. The MiniLMV2 paper introduced multiple variants, all having various sizes and various teacher models. Among them, we chose the L6-H768 variant which demonstrated the best performance across a wider variety of tasks. We also experimented with a tiny DeBERTa variant trained on QNLI and SNLI datasets which were introduced in the paper by He et al. [12]. In their paper, the authors retrained various smaller versions of DeBERTa using a variant of embedding sharing [5]. We have also experimented with another BERT-based model dubbed RoBERTa-base [21]. This version of the RoBERTa-base model was also trained on the QNLI [35] and SNLI [2] datasets. Additionally, we did some experiments with simple ensemble stacked models. For the ensemble classifiers, we experimented with Random Forest [14] and Voting.

## Our Method-Task 1

### Task Description

The Case Law Retrieval Task in COLIEE investigates the performance of systems that search a set of case law records that support the unseen case law. The goal of the task is to return “noticed cases” in the given collection to a query. A case is “noticed” by a query case if and only if the case is referenced by the query case. In this task, the explicit references to other cases are redacted from the query case contents.

### Dataset Analysis

The Task 1 corpus is composed of Federal Court of Canada case laws, and contains all query and noticed cases in a single pool. The training data contains labels indicating which cases are noticed by each query case. In the test data, only the query cases are given and the task is to predict which cases should be noticed for each of the test query cases.

In Task 1, the training dataset consists of 4400 unique files (cases), with 959 of those identified as query cases. There are a total of 4488 noticed cases, an average of 4.67 noticed cases per query case (a case in the pool may be referenced by more than one query case, so 4488 noticed cases include the same cases multiple times.). In the provided test dataset there were 319 query cases and a total of 1335 files (cases).

Each case in the pool is a text file containing the full contents of a legal case. Apart from these files, the training data contains a JSON file with the query case list, and for each query case the list of noticed cases. The test data contains the pool of test files and a JSON file with the list of query cases. A fragment of the training JSON file is given below as an example:

"008447.txt": ["072495.txt","082291.txt","004851.txt"],

"067501.txt": ["038025.txt"],

"007627.txt": ["003575.txt","043211.txt"],

...

Where you can see the list of query cases (008447.txt, 067501.txt, and 007627.txt), and the respective list of noticed cases for each query case. For example, if 008447.txt and a pool of cases are given as input, the output should be the noticed cases from 008447.txt, which are 072495.txt, 082291.txt, and 004851.txt in this example. All the cases in the training JSON are given in the training pool, which also contains cases that are not referenced by any query case. The same happens in the test dataset: all the given query cases, and all the noticed cases (which need to be found by the participants) are included in the test pool.

### Details of our Approach

In case law, a “noticed” case is a precedent cited in a case, the precedent being considered somewhat relevant to that case at hand. It is very hard to model why a precedent may be seen as relevant to a given case, for it is a very subjective consideration by the judge while working on the case. It may be that the judge found a similar argument in a previous case, or that the previous case establishes the jurisprudence for the legal issue under consideration, or that a case is noticed because it actually contradicts a point raised by one of the parties and the judge is showing that contradiction, etc. In this paper, we considered semantic similarity between two cases to be a reasonable proxy to determine whether a case should be noticed, and develop our method starting from that hypothesis.

Our approach to the case law retrieval task relies on the use of a sentence-transformer model to generate a multidimensional numeric representation of text. This model is applied to each paragraph from both the query case and every candidate case. We then use a cosine measure to determine the distances between the 768-dimension vectors from the query paragraphs and the candidate paragraphs. A 10-bin histogram of those distances is generated and a Gradient Boosting [8] binary classification model is trained on those inputs.

Given the formulation of the problem, we had to make some choices to produce a manageable training dataset: since the test set contains a total of approximately 1300 cases of which 319 are query cases, we assumed we should generate a training dataset with around 1000 negative samples per query case. So we needed to down-sample the negative samples in the training dataset to 1000. At the same time, the positive class is significantly underrepresented (less than 5 samples per query case on average), so we over-sampled those examples by simple replication.

We also implemented some simple pre-processing steps:

- *Removal of French contents* through a language identification model based on a naive Bayesian filter [22];
- *Splitting of input text into paragraphs* based on simple pattern matching which relies on the common format used in cases. This method relies on finding a sequence of numbered paragraphs (specified as digits between brackets) as the first characters in the line starting at “[1]” and looking for the next natural number;
- *Extraction of dates mentioned in the cases* is done by the application of a named entity recognition model [15]. These dates are used to remove candidate cases that mention dates more recent than the most recent date mentioned in a query case, under the assumption those candidates cannot be a true noticed case because they are more recent than the query case. So, we extract all date entities in both the query and the candidate case, then, if the query case contains a date that is more recent than the most recent date in the candidate case, that candidate case will be removed from the list.

At inference time we do the following steps:

- *Date filtering:* we apply the same date pre-processing steps mentioned above;
- *Histograms*: we generate histograms for every pair of query documents and each candidate that does not contain dates more recent than the query document dates;
- *Apply model:* we use those histograms as inputs to build our classification model.

Based on our analysis of the training dataset, we also apply some simple post-processing steps:

- *Number of noticed cases per query case:* the average number of noticed cases per query case in the training dataset is 4.67, so we establish a range of 3 to 10 maximum noticed cases per query case;
- *Confidence score:* we establish a minimum confidence score for the classifier, disregarding outputs that are below a given threshold;
- *Repeating noticed cases:* if the same case is noticed across many different query cases, we also remove that noticed case from our final answer as it is observed in the training dataset that this is an uncommon situation.

**Fig. 1:**
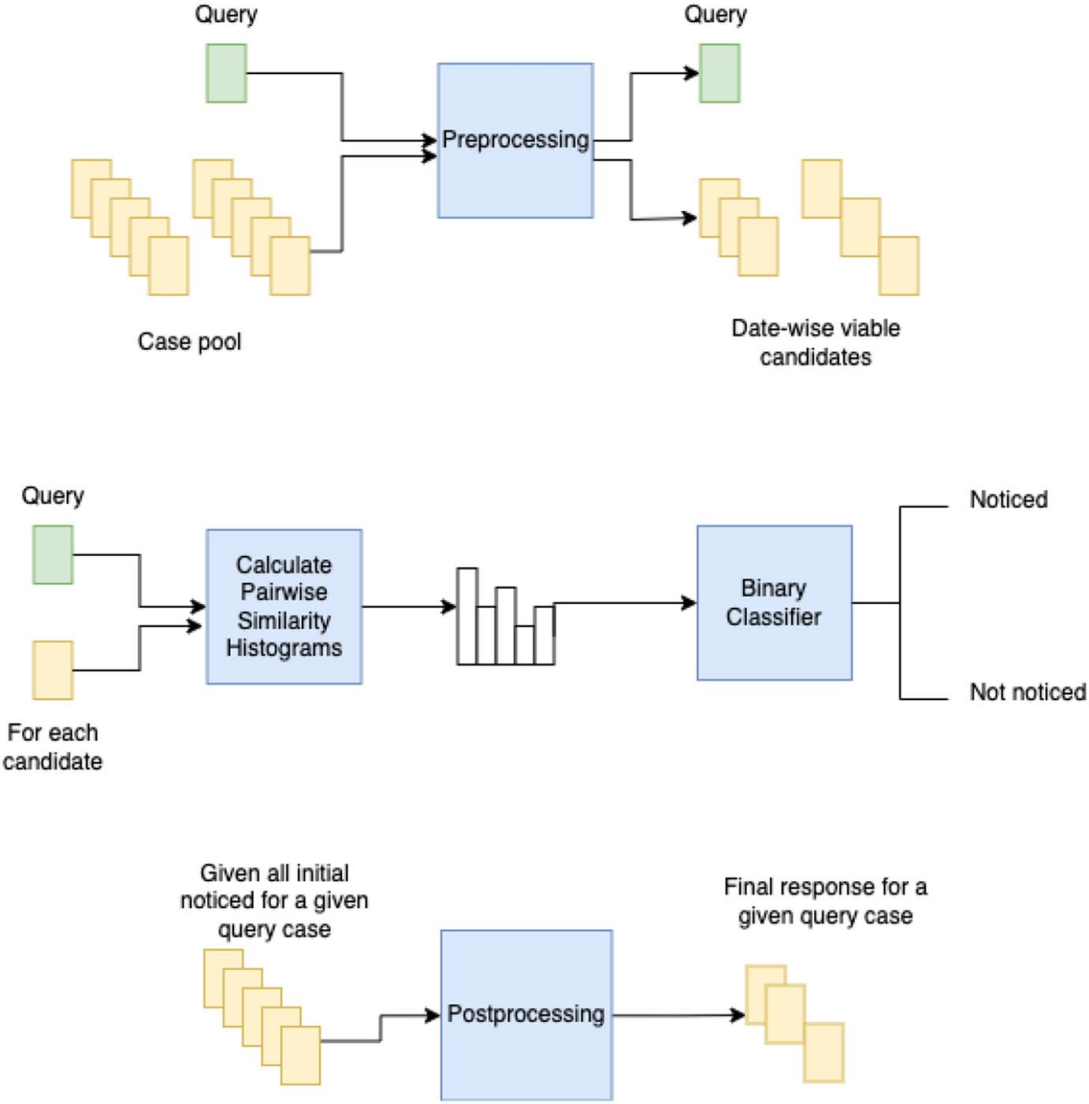
High-level diagram for the Task 1 approach

A high-level diagram of the approach is given in Fig. 1. In the figure, you can see the pre-processing, histogram generation, binary classification, and post-processing that have been explained above.

We have experimented with a range of parameters for each one of those post-processing criteria and selected the 3 combinations that produced the best output in a validation set containing 50 query cases.^2^

#### Sentence-Transformer Model

The model used to produce the 768-dimensional representations for the case paragraphs was the HuggingFace sentence-transformers/all-mpnet-base-v2 model.^3^ That model was trained on large sentence-level datasets using a self-supervised contrastive learning objective, which used the pre-trained Microsoft/ mpnet-base model.^4^ Note that the original authors use a contrastive learning objective: given a sentence from the pair, the model should predict which of a set of randomly sampled other sentences was paired with it in the dataset.

#### Binary Classification Model

We used the Gradient Boosting model [8] to train our binary classification model, which was trained on the calculated similarity histograms as described above. Since the training dataset is significantly unbalanced, we over-sample the positive class by simple duplication and under-sample the negative class by establishing a target maximum number (which was chosen as 1000 samples). The only hyper-parameter we varied in the classifier itself was the number of estimators, which was set to 1000, 3000, and 5000.

#### Hyper-Parameter Setting

We performed a grid search for 3 hyper-parameters:

- Maximum number of noticed cases per query case: based on the dataset analysis performed, given the average number of noticed cases per query case in the training set is around 5, we experimented with establishing a limit that varied from 3 to 10 (step 1) in an attempt to reduce the false positives;
- Minimum confidence score: we trained a binary classifier to determine if a given case should be noticed concerning a given query case. With this hyper-parameter, we can filter candidate cases for which the classifier confidence score is below a given threshold. We experimented with values from 0.55 to 0.80 (step 0.05);
- Maximum duplicate noticed cases: we noticed in our validation results that the same case was classified as noticed for more than one query case, which is not common in the training dataset, so we established the maximum number of times the same case can be present in the output. This parameter was varied from 1 to 5 (step 1).

The 3 best performing hyper-parameter combinations were used in our COLIEE submission. You can see that the best (max noticed cases = 10, min score = 0.8, max dups = 3) achieved good precision but poor recall. The second-best combination (9, 0.7, 2) had even higher precision, but very poor recall. We attribute this to the effect of the minimum confidence score, which was higher in this case, whereas the other parameters were similar. Even though the difference in the final f1-score was not material, having the ability to tweak parameters and influence precision and recall would be a good feature of the method in real-world applications, where users could adopt parameters according to their requirements concerning precision and recall.

**Table 1:** Official results for the Case Law Retrieval task. Prec. is an abbreviation for precision

Team	F1	Prec.	Recall	Team	F1	Prec.	Recall
THUIR	0.3001	0.2379	0.4063	UFAM	0.2545	0.2975	0.2224
THUIR	0.2907	0.2173	0.4389	JNLP	0.2511	0.1971	0.3458
IITDLI	0.2874	0.2447	0.3481	JNLP	0.2493	0.1931	0.3516
THUIR	0.2771	0.2186	0.3783	OurApproach	0.2390	0.3045	0.1967
NOWJ	0.2757	0.2263	0.3527	OurApproach	0.2345	0.2400	0.2293
NOWJ	0.2756	0.2272	0.3504	UFAM	0.2345	0.3199	0.1851
IITDLI	0.2738	0.2107	0.3912	UFAM	0.2156	0.3182	0.1630
IITDLI	0.2681	0.2063	0.3830	YR	0.1377	0.1060	0.1967
JNLP	0.2604	0.2044	0.3586	YR	0.1051	0.0809	0.1502
NOWJ	0.2573	0.2032	0.3504	LLNTU	0.0000	0.0000	0.0000
OurApproach	0.2555	0.2847	0.2317	LLNTU	0.0000	0.0000	0.0000

## Our Method-Task 4

In Task 4, the problem of answering a legal yes/no question can be viewed as a binary classification problem. We assume that a set of questions *Q*, where each question $q_{i} \in Q$ is associated with a list of corresponding article sentences $a_{i1}, a_{i2},$...$, a_{im}$, where $y_{i} = 1$ if the answer is “yes” and $y_{i} = 0$ otherwise. Therefore, our task is to learn a classifier that can predict the entailment answers of any question-article pairs. BERT [6] has shown good historical performance in both COLIEE and in general on the natural language inference tasks. However, Jiang and Marnaffe [16] insisted that despite high F1-scores, BERT models have systematic error patterns, suggesting that they still do not capture the full complexity of human pragmatic reasoning.

**Fig. 2:**
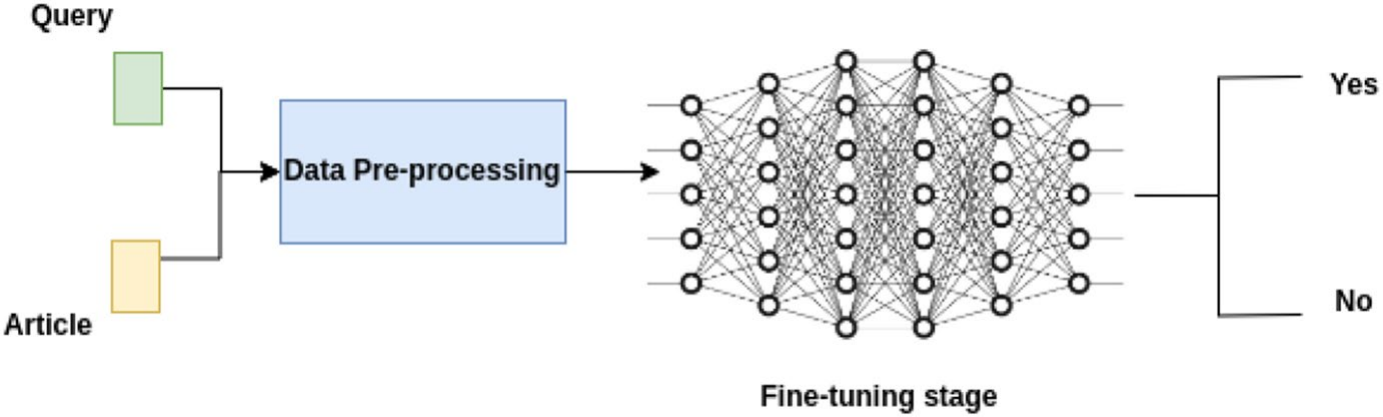
High-level diagram for the Task 4 approach. The inputs are the queries and articles. We first pre-process the data, and then for the downstream tasks, we fine-tune a deep network to predict the output (yes vs no)

We reformulate the problem as a natural language inference task, where the objective of the model is to determine the logical relationship between a premise and a hypothesis (e.g., whether the hypothesis entails, contradicts, or is neutral with respect to the given premise). In the case of answering a legal yes/no question, if the NLI model predicts the relationship as entailment, we then consider the prediction as “yes” otherwise “no.” A high-level diagram is shown in Fig. 2.

To construct the training data for the NLI model fine-tuning, we modify the ground-truth labels. For questions with a ground-truth label of “yes,” we change them to “entailment,” and for questions with a label of “no,” we change them to “contradiction.” In the pre-processing step, we also convert the inputs of the data to lowercase. Because we have two inputs before making a prediction, we follow the procedure proposed by [27], i.e., we concatenate the sentence embedding *u* and *v* from input 1 (query) and 2 (article) respectively, and then use the element-wise difference $|u - v|$ and multiply it with the trainable weight *W*.

We then fine-tune the model by minimizing the cross-entropy loss over the labeled training data to penalize incorrect classification.

**Table 2:** NLI (Task 4) results on test data

Team	sid	Correct	Accuracy
	BaseLine	No 52/All 101	0.5149
JNLP	JNLP3	79	0.7822
JNLP	JNLP1	76	0.7525
JNLP	JNLP2	76	0.7525
KIS	KIS2	70	0.6931
KIS	KIS1	68	0.6733
Our Approach	Our_V2	67	0.6634
AMHR	AMHR01	66	0.6535
KIS	KIS3	66	0.6535
AMHR	AMHR03	65	0.6436
LLNTU	LLNTUdulcsL	63	0.6238
Our Approach	Our_V1	63	0.6238
HUKB	HUKB2	60	0.5941
CAPTAIN	CAPTAIN.gen	59	0.5842
CAPTAIN	CAPTAIN.run1	58	0.5743
LLNTU	LLNTUdulcsS	57	0.5644
HUKB	HUKB1	56	0.5545
HUKB	HUKB3	56	0.5545
LLNTU	LLNTUdulcsO	56	0.5545
NOWJ	NOWJ.multi-v1-jp	55	0.5446
CAPTAIN	CAPTAIN.run2	53	0.5248
NOWJ	NOWJ.multijp	53	0.5248
NOWJ	NOWJ.multi-v1-en	49	0.4851

## Results

### Task 1 Results

The results of the official COLIEE evaluation set are shown in Table 1:

Our best result was achieved with the following post-processing parameters: minimum confidence score = 0.80, maximum noticed cases = 10, maximum number of repeated noticed cases^5^ = 3. Our second-best score had similar parameters (0.7, 9, and 2, respectively). In the third submission we used 0.65, 10, and 3, respectively). This provided a more balanced trade-off between precision and recall, as opposed to the first two which had a higher precision but a lower recall. This is an interesting characteristic for real-world applications, as one could make an informed decision on how to tweak parameters depending on which metric is more important for their particular scenario.

Ideally, we would perform a thorough error analysis to understand the main areas/cases of improvement for our approach, but that kind of error analysis is not feasible in this scenario as it would require reading every case in the dataset, potentially multiple times, and figure out what characteristics in the inputs are not well modeled in the approach.

### Task 4 Results

Table 2 shows the Task 4 results on test data in COLIEE 2023. We submitted two results, $Our\_V1$ fine-tuned on DeBERTa-small [13] and $Our\_V2$ fine-tuned on DeBERTa-large model [27]. In the table, we report the performance of the best model i.e., $Our\_V2$. The test results considering only one best system in each team are in Table 3:

**Table 3:** NLI (Task 4) results on test data considering only the best system in each team

Team	sid	Correct	Accuracy
	BaseLine	No 52/All 101	0.5149
JNLP	JNLP3	79	0.7822
KIS	KIS2	70	0.6931
Our Approach	Our_V2	67	0.6634
AMHR	AMHR01	66	0.6535
LLNTU	LLNTUdulcsL	63	0.6238
HUK	HUKB2	60	0.5941
CAPTAIN	CAPTAIN.gen	59	0.5842
NOWJ	NOWJ.multi-v1-jp	55	0.5446

The DeBERTa-large model achieved an accuracy of 55.77% for the “no” class and 77.55% for the “yes” class. We found that the current model struggles in predicting the correct class “no” which understandably requires a deeper understanding of the semantics of the input.

### Further Experiments for Task 4 with Smaller NLI Models

In our experiments with Task 4 during the COLIEE competition, we got the best results in terms of accuracy with DeBERTa-large [12]. However, one of the challenges with larger models is fine-tuning them for downstream tasks (re-purposing them on a specific task); the computation complexity is higher due to the higher number of parameters. Moreover, for NLI, there is a prior evidence that smaller BERT-based models perform just as well or better [12]. So in this section, we present some subsequent experiments with smaller NLI models as well as traditional ensemble models to see how these relatively simpler models compare with DeBERTa-large.

For our experiment, we picked two types of models, one for knowledge distillation called nli-MiniLM2-L6-H768 (MiniLMV2) [32], and the other type would be smaller variants of BERT-based models such as DeBERTa and RoBERTa [21]. Since DeBERTa-large had the best performance in our initial experiments, we chose all 3 smaller variants, namely DeBERTa-xsmall, DeBERTa-small, and DeBERTa-base. From RoBERTa we pick the base model, i.e. RoBERTa-base. All 5 of these models were trained on QNLI and SNLI datasets. Finally, we also did some experiments with ensembling on these 5 smaller transformer models. For the ensemble classifiers, we experimented with Random Forest [14] and Voting. The results are in Table 4.

**Table 4:** NLI (Task 4) Further Experiments for Task 4 with smaller NLI models

Model Name	Parameters (M)	Accuracy
Our_V2 [DeBERTa-large]	304	**0**.**66**
DeBERTa-xsmall	22	*0.65*
DeBERTa-small	44	0.63
MiniLM2	81	0.6
DeBERTa-base	86	0.62
RoBERTa-base	125	0.6
Stacking-RandomForest	N.A	**0**.**66**
Stacking-Voting	N.A	**0**.**7**

The results in Table 4 show that smaller models do fairly well compared to DeBERTa-large. However, it also seems that their performance is *not* a simple function of their size. For example, DeBERTa-xsmall performs better compared to DeBERTa-small despite being the smaller model. This performance conforms to prior work [12] where DeBERTa-xsmall achieved better results than DeBERTa-small on an NLI task.

Finally, ensemble models perform better than the individual small transformers. An Ensemble model with a Random Forest as the meta-classifier performs better than any of the individual smaller transformer models and the Voting meta-classifier with all 5 smaller transformers performs better than DeBERTa-large.

Ensemble models likely work better than individual models because those models individually differ from one another. DEBERTa-base, MiniLM2, and DeBERTa-small predict the “yes” (entailment) class better and struggle with the “no” class. DeBERTa-xsmall has equal performance in predicting the “yes” and “no” classes with slightly better performance for the “yes” class. Finally, the RoBERTa-base model achieves similar performance in predicting both classes with slightly better performance for the “no” class. Overall, this mix of different models seems to help with the ensemble models’ performance.

### Further Experiments for Task 4 with Legal-BERT Models

We also conducted another set of experiments with Legal-BERT [4]. Legal-BERT is a BERT model that has been augmented by pre-training with legal data and has been shown to achieve state-of-the-art results on some challenging tasks in the legal domain. Legal-BERT has 5 variants.^6^ The Contracts model is pre-trained on the SEC-EDGAR repository, containing 76,366 United States (US) contracts. The EURLEX model is pre-trained on the EURLEX repository containing 19,867 cases from the European Court of Justice (ECJ). The ECHR model is pre-trained on 12,554 cases from HUDOC, the repository of the European Court of Human Rights (ECHR). Finally, the base and the small model are pre-trained on all of the aforementioned repositories. As the names suggest, the base and small models are meant to be more general whereas the other 3 models are aimed to be geared towards legal documents of specific jurisdictions and types.

For Legal-BERT there is a mismatch between the corpus that it was pre-trained on and the data it is being fine-tuned on. As mentioned before, Legal-BERT and its variants are pre-trained on the US and European Union (EU) legislation corpora. On the other hand, the Task 4 data is based on the Japanese bar exam corpus, translated into English. We conduct our experiments on Legal-BERT variants. Because we believe that despite being pre-trained on corpora that are from different jurisdictions, some commonalities do exist and the remaining mismatch can be addressed by fine-tuning. Furthermore, the lack of volume in the Japanese law corpora precludes us from pre-training a transformer for our requirements. We leave the generation of a sufficient volume of corpora related to Japanese law and the subsequent pre-training of a transformer model for future work.

Here we report results on 4 Legal-BERT variants, Contracts, EURLEX, ECHR, and Base. We consider Task 4 as a binary classification problem (entailment or no entailment) and train Legal-BERT classifiers. The results are in Table 5. From the table, we can see, that Legal-BERT-ECHR has the best performance among the Legal-BERT variants. As these models are pre-trained on various types of documents, another future work of interest would be to investigate whether a particular type of legal document during pre-training helps discern the entailment relationship between two bodies of text.

**Table 5:** NLI (Task 4) results with Legal-BERT models

Model Name	Accuracy	Model Name	Accuracy
Legal-BERT-base	0.5	Legal-BERT-ECHR	0.58
Legal-BERT-Contracts	0.52	Legal-BERT-EURLEX	0.5

### Error Analysis

We selected three models for error analysis: DeBERTa-large, Stacking-Voting, and Legal-BERT-ECHR, as they demonstrated the best performances within their respective model groups. Among the DeBERTa models that we employed, DeBERTa-large showed the highest accuracy. Stacking-Voting outperformed the other ensemble model that we used. Additionally, among the Legal-BERT models we employed, Legal-BERT-ECHR demonstrated the best performance.

We categorized error types from unsuccessful instances, as presented in Table 6. Legal-BERT-ECHR had the highest number of errors when it predicted entailment outputs for queries related to problems with resolving pronoun references. One example is as follows:

**Table 6:** Error analysis of the models

Error type	Count of	Wrong	Prediction
	DeBERTa	Stacking	Legal-BERT
	-large	-Voting	-ECHR
Incorrect reference of pronoun	14	11	21
Incorrect negation/antonym detection	6	7	7
Incorrect analysis of paraphrases	2	3	1
Incorrect analysis of exceptions	2	2	2
Incorrect analysis of conjunction(and,or)	1	1	1
Incorrect assessment of condition	7	5	8
Incorrect interpretation of others	2	1	2

- **Input**
  - **[Input Article]** Article 178: The transfer of a real right on movables may not be duly asserted against a third party unless the movables are delivered. Article 184: If a thing is possessed through an agent, the principal orders that agent to thenceforward possess that thing on behalf of a third party, and that third party consents thereto, the third party acquires the possessory rights.
  - **[Input Query]** A sold Painting X, owned by A, to C while depositing it with B and ordered B to thenceforward possess X on behalf of C, and B consented thereto. In this case, C may duly assert the acquisition of the ownership of X against any third parties.
- **Output: No**

To solve the example, a model needs to solve what each pronoun refers to. In the example, “A” and “B” in the hypothesis refer to “principal” and “agent” in the premise, respectively. “X” and “C” in the hypothesis refer to “thing” and “third party” in the premise.

Out of the models, only Stack-Voting correctly predicted the entailment in the example, while Legal-BERT-ECHR and DeBERTa-large made incorrect predictions.

Due to the limited number of test samples, a thorough examination of error analysis was constrained. Nevertheless, based on the available 101 test samples, Stacking-Voting demonstrated the highest accuracy in addressing queries related to resolving pronoun references and analyzing the condition of legal sentences. Here is an example that demonstrates the significance of accurately analyzing a condition.

- **Input**
  - **[Input Article]** Article 126: The right to rescind an act is extinguished by the operation of the prescription **if it is not exercised within five years from the time when it becomes possible to ratify the act.** The same applies if 20 years have passed from the time of the act.
  - **[Input Query]** The right to rescind an act is extinguished by the operation of the prescription **if it is not exercised within five years from the voidable act.**
- **Output: No**

In the example, both the legal article and the query contain the “if” condition and the corresponding conclusion. While the condition matches in both the article and query, the “if” condition differs. Therefore, the correct entailment label should be “no”. Stacking-Voting made the correct prediction.

Legal-BERT-ECHR showed the fewest errors when answering the queries related to paraphrasing. This could be because Legal-BERT-ECHR was pre-trained on legal corpora, whereas the other two models were pre-trained on general domain corpora. Following is an example that shows paraphrasing between the article and query:

- **Input**
  - **[Input Article]** Article 449: If a guarantor that guarantees an obligation which may be voidable due to the principal obligor’s limited capacity to act, is aware, **at the time of entering into a guarantee contract,** of the cause for its voidability, that guarantor is presumed to have assumed an independent obligation of the same subject matter in the event of non-performance by the principal obligor or rescission of the obligation.
  - **[Input Query]** If a guarantor that guarantees an obligation which may be voidable due to the limited capacity to act, is aware, **at the time of conclusion of the guarantee contract,** of the cause for its voidability, that guarantor is presumed to have assumed an independent obligation of the same subject matter in the event of non-performance by the principal obligor or rescission of the obligation.
- **Output: Yes**

In this example, the phrase “at the time of entering into a guarantee contract” was paraphrased as “at the time of conclusion of the guarantee contract” in the query. Legal-BERT-ECHR made a correct prediction, while Stacking-Voting made an incorrect prediction for this example. Meanwhile, DeBERTa-large excelled in handling queries that needed negation or antonym detection. One example that requires negation/antonym detection is as follows:

- **Input**
  - **[Input Article]** Article 354 If the claim of a pledgee of movables is not satisfied, the pledgee may make a request to the court seeking the immediate appropriation of the thing pledged for the satisfaction of that claim **in accordance with the evaluation of an appraiser** only when there are reasonable grounds. In this case, the pledgee of movables must notify the obligor in advance of the request.
  - **[Input Query]** If the claim of a pledgee of movables is not satisfied, the pledgee may immediately appropriate the thing pledged for the satisfaction of that claim by getting the permission of the court, **instead of getting the evaluation of an appraiser,** only when there are reasonable grounds for not getting the evaluation of an appraiser.
- **Output: No**

To solve the example, a model needs to understand that “instead of getting the evaluation of an appraiser,” means “NO evaluation of an appraiser”, which contradicts the premise. Out of the three models, only DeBERTa-large made a correct entailment prediction for this example.

To facilitate error analysis, we assigned each incorrectly predicted sample to one category in Table 6. In cases where a sample exhibited multiple error types, we assigned it to the category representing the most challenging aspect for correct analysis. Large language models are black-box models, making it challenging for us to comprehend and explain the entailment prediction process in a way that is understandable to humans. Therefore, our categorization in Table 6 is based on human interpretation, and it has the limitation that these categories may not precisely align with the true behavior of each model. As part of our future work, we plan to employ explainable AI techniques capable of providing faithful insights into the rationale behind each model’s entailment predictions.

In future work, we aim to develop representations that can be used as proxies to assist deep nets in better comprehending legal concepts. In addition, we believe that integrating legal expert knowledge into large language models is crucial for addressing the failures of the large language models. We will conduct a comprehensive exploration of how legal expert knowledge can be effectively integrated into these models. This integration will assist in connecting pronouns in queries to legal terms in law articles, understanding complex conditions, and interpreting intricate conjunctions.

## Conclusion

We have explained our use of various language models for legal entailment and question-answering in COLIEE 2023. For the case law retrieval task (Task 1), we used a sentence-transformer model to generate a multidimensional numeric representation of text, with some heuristic pre-processing and post-processing methods. For the statute law tasks, our transformer-based NLI system was ranked 3rd in Task 4. Furthermore, we have conducted supplementary analyses and experimented with various approaches, observing a slight enhancement in terms of predictive accuracy when using an ensemble model. A limitation of our method for Task 1 is that, although we calculate pairwise similarities between paragraphs to create histograms, it still considers similarity between whole documents. Through a more detailed data analysis, we notice that references to other cases are made in smaller contexts. Thus, we want to explore methods that identify similarities (or more broadly, relevance) in short contexts as opposed to whole documents. At the implementation level, we also want to explore open-source LLMs that can be used in a controlled environment and whose pre-training and fine-tuning details are known and do not interfere with the competition’s terms. In addition, for task 4, it is essential to investigate techniques to augment the training data and find effective techniques for learning representations of legal texts. We aim to develop representations that can be used as proxies to assist deep nets in better comprehending legal concepts and thus improving the discriminative power. Moreover, it is also crucial to explore additional quantitative metrics that could help in assessing the strength of representations encoded by deep nets before training for the downstream tasks.

## Footnotes

1 https://sites.ualberta.ca/~rabelo/COLIEE2023/.

2 The validation set was randomly drawn from the provided dataset and has no overlap with the cases used for training.

3 https://huggingface.co/sentence-transformers/all-mpnet-base-v2.

4 https://huggingface.co/microsoft/mpnet-base?text=The+goal+of+life+is+%3Cmask%3E.

5 We simply remove noticed cases that appear in more than the maximum allowed query cases. An obvious improvement is to keep just the highest-scoring noticed cases.

6 https://huggingface.co/nlpaueb/legal-bert-base-uncased.
